# Unexpected conformational variations of the human centromeric chromatin complex

**DOI:** 10.1101/gad.307736.117

**Published:** 2018-01-01

**Authors:** Jitendra Thakur, Steven Henikoff

**Affiliations:** Howard Hughes Medical Institute, Basic Sciences Division, Fred Hutchinson Cancer Research Center, Seattle, Washington 98109, USA

**Keywords:** centromeres, chromatin, methodology

## Abstract

We combined classical salt fractionation with chromatin immunoprecipitation to recover human centromeric chromatin under native conditions. We found that >85% of the total centromeric chromatin is insoluble under conditions typically used for native chromatin extraction. To map both soluble and insoluble chromatin in situ, we combined CUT&RUN (cleavage under targets and release using nuclease), a targeted nuclease method, with salt fractionation. Using this approach, we observed unexpected structural and conformational variations of centromere protein A (CENP-A)-containing complexes on different α-satellite dimeric units within highly homogenous arrays. Our results suggest that slight α-satellite sequence differences control the structure and occupancy of the associated centromeric chromatin complex.

## Abstract (precis)

Thakur and Henikoff combined CUT&RUN, a targeted nuclease method, with salt fractionation and observed unexpected structural and conformational variations of centromere protein A (CENP-A)-containing complexes on different α-satellite dimeric units within highly homogenous arrays.

The fidelity of chromosome segregation depends on the efficient capture of chromosomes by spindle microtubules via proteinaceous kinetochores, which assemble at specific chromosomal loci called centromeres. Human centromeres comprise 0.5- to 5-Mb-long tandem arrays of an ∼170-base-pair (bp) α-satellite repeat unit (Alexandrov et al. 2001). α-Satellite DNA originated in the primate lineage and has since evolved by repeat expansion, resulting in highly homogenous young arrays at the core, with more diverged α-satellite sequences occupying centromere edges. Assembly of these homogenous α-satellite arrays into contiguous maps has presented a serious challenge to existing sequence assembly technologies. To address this problem, we recently used bottom-up hierarchical clustering of sequences bound by centromere proteins for de novo identification of functional centromeric α satellites. We found that the most abundant α-satellite arrays contain a basic 340-bp or 342-bp dimeric unit (Henikoff et al. 2015), which belongs to previously characterized SF1 and SF2 suprachromosomal families of α satellites, respectively (Alexandrov et al. 2001).

Centromeric α satellites are included in specialized chromatin, where canonical histone H3 is replaced by its cenH3 variant, called centromere protein A (CENP-A) (Palmer et al. 1987; Fukagawa and Earnshaw 2014). CENP-A is part of the constitutive centromere-associated network (CCAN) complex, which includes CENP-B, CENP-C, CENP-N, CENP-T, CENP-W, CENP-S, and CENP-X (Hori et al. 2008). Using a comparative chromatin immunoprecipitation (ChIP) with DNA sequencing (ChIP-seq) strategy that included native ChIP (N-ChIP), cross-linking ChIP (X-ChIP), and sequential ChIP (ReChIP), we showed previously that CENP-B, CENP-C, and CENP-T are physically integrated and form a coherent complex with CENP-A nucleosomes. Micrococcal nuclease (MNase) digestion of CENP-A, CENP-C, and CENP-T X-ChIP resulted in >165-bp protection over α-satellite dimers (Thakur and Henikoff 2016), whereas under native conditions, MNase digestion resulted primarily in shorter CENP-A-bound α-satellite fragments ranging from ∼100 to ∼135 bp (Hasson et al. 2013; Henikoff et al. 2015; Nechemia-Arbely et al. 2017).

We and others have found that centromeric chromatin is stable when extracted with 350–500 mM NaCl (Zhang et al. 2012; Hasson et al. 2013; Henikoff et al. 2015). We also found that 500 mM NaCl increased the recovery of centromeric chromatin relative to low-salt conditions (Thakur and Henikoff 2016), raising the question of whether the differences in recovery reflect qualitative differences in the nature of centromeric chromatin. As classical chromatin salt fractionation has been used to separate nucleosomes with different physical properties (Sanders 1978), functions (Rocha et al. 1984), and genome-wide distributions (Henikoff et al. 2009; Jahan et al. 2016), we wondered whether most of the centromeric chromatin had been rendered insoluble by the presence of CCAN components that are absent from the soluble fraction that is typically recovered in native MNase-ChIP studies.

To address the possibility that differential solubility under native conditions reflects qualitative differences in centromeric chromatin, we subjected salt-fractionated chromatin to N-ChIP of centromeric proteins. We further explore differences in salt solubility by adapting our recently developed CUT&RUN (cleavage under targets and release using nuclease) in situ targeted mapping method for profiling specific centromeric components. We found that minor sequence differences between dimeric repeats belonging to the same α-satellite subfamily correspond to differences in both centromere protein binding and the structure of the complex itself.

## Results and Discussion

### Centromeric chromatin is insoluble under low-salt conditions

**Figure 1.**
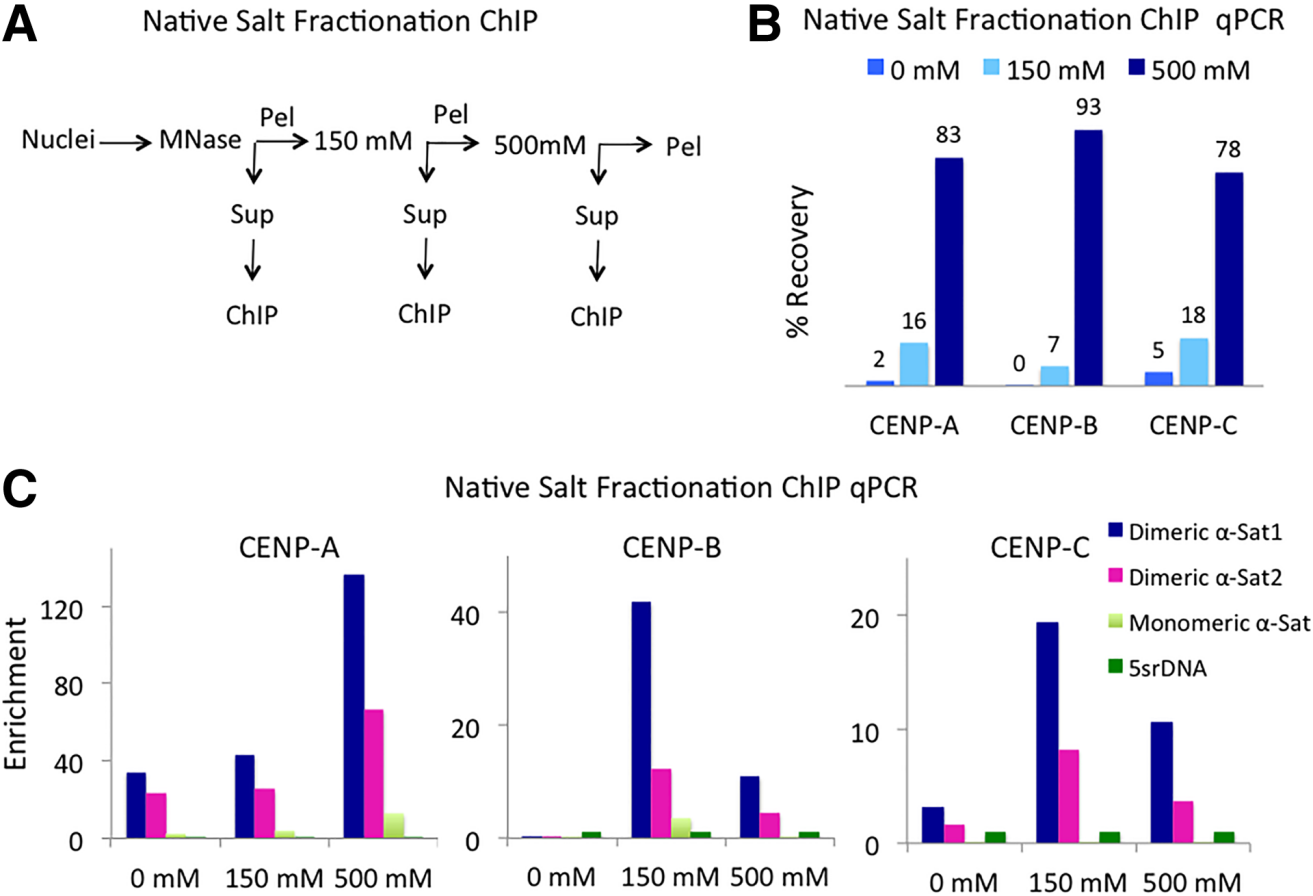
Most CCAN complexes are highly insoluble. (*A*) Native salt fractionation ChIP scheme. (*B*) Percent recovery in various salt fractions as determined by qPCR on CENP-A, CENP-B, and CENP-C salt-fractionated N-ChIP DNA using centromeric α-satellite primers. (*C*) The enrichment of centromeric α-satellite dimers and noncentromeric monomeric α satellites was calculated as the ratio of α satellites to 5srDNA in various salt fractions. Primer sequences (Supplemental Table 2) were derived from two centromeric α-satellite dimers and one α-satellite monomer.

To analyze the structure and positioning of CENP-A nucleosomes associated with soluble and insoluble chromatin, we digested nuclei with MNase and then successively extracted the material using buffers containing 0–500 mM NaCl (Fig. 1A). Each soluble fraction was then subjected to N-ChIP using antibodies against CENP-A, CENP-B, and CENP-C. We analyzed the percent recovery of centromeric chromatin in each N-ChIP salt fraction by quantitative PCR (qPCR) using immunoprecipitated DNA as the template. Of the total centromeric chromatin extracted, only ∼2% and ∼15% of the total N-ChIP DNA was recovered in the no-salt and low-salt fractions, respectively, whereas the remaining >80% was recovered in the high-salt (500 mM) fraction (Fig. 1B). All three salt fractions showed enrichment over centromeric α-satellite dimers relative to noncentromeric α-satellite monomers (Fig. 1C). Such low recovery of chromatin in no-salt and low-salt fractions is consistent with classical studies in which salt fractionation was used to define active chromatin (Sanders 1978; Rocha et al. 1984). Thus, the large majority of centromeric CENP-A chromatin and associated CCAN complexes, such as bulk chromatin, is insoluble under low-salt conditions. Enrichment of CENP-A chromatin in the high-salt fraction is in contrast to enrichment of H3.3 and H2A.Z “active” histone variants in the low-salt fraction (Henikoff et al. 2009). We conclude that the physical properties of nucleosomes are also reflected in chromatin states defined by histone variants.

### CCAN complexes extracted with high salt are heterogeneous in size

We showed previously that high-resolution X-ChIP using antibodies against CENP-A, CENP-C, and CENP-T recovers fragments >165 bp in size (Thakur and Henikoff 2016). We wondered whether the relative lack of large fragments obtained using N-ChIP in other published studies (Lacoste et al. 2014; Henikoff et al. 2015; Nechemia-Arbely et al. 2017) might have resulted from the failure to solubilize most centromeric chromatin under low-salt conditions. To address this possibility, we prepared Illumina sequencing libraries from N-ChIP salt fractions and subjected them to paired-end sequencing. We sequenced 250 bases on both ends of each fragment and merged overlapping pairs (Henikoff et al. 2015). In this way, we determined fragment lengths directly rather than relying on mapping of fragment ends to specific sequences, which can be ambiguous for homogeneous tandem repeats.

**Figure 2.**
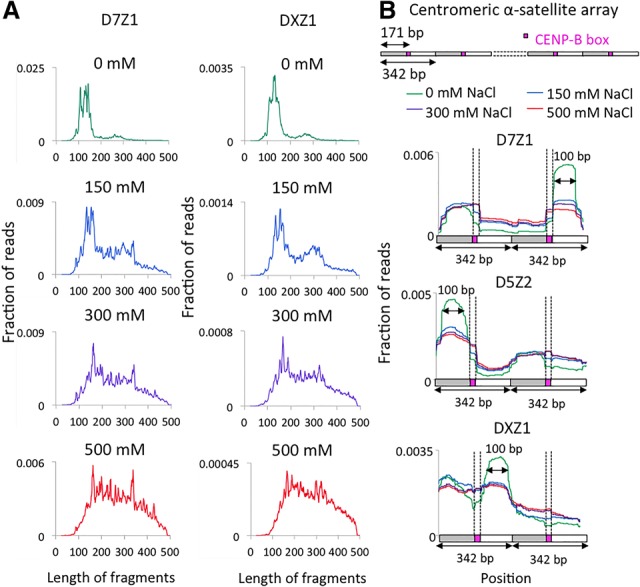
Heterogeneous protection of centromeric α satellites in N-ChIP. (*A*) Fragment length analysis of 250-bp × 250-bp merged pairs obtained from native salt fractionation ChIP-seq data sets on D7Z1 (*top*) and DXZ1 (*bottom*) in various fractions. (*B*, *top* panel) An example of a contig containing dimeric α-satellite units arranged in tandem. CENP-A profiles were generated by mapping merge pairs to D7Z1 and DXZ1 arrays. (*Bottom* three panels) A region spanning two tandem 340-bp dimers from the contigs is presented. Magenta boxes represent CENP-B boxes.

We mapped merged pairs to sequence contigs consisting mostly of dimeric α-satellite units, which had been shown previously to occupy 20 of 24 human centromeres (Alexandrov et al. 2001; Henikoff et al. 2015). We focused on contigs representing subfamilies that had been shown to be centromeric (D5Z2, D7Z1, and DXZ1) by combined fluorescence in situ hybridization and CENP-A immunofluorescence and endpoint ChIP-qPCR with a CENP-A antibody (Slee et al. 2012). We observed striking differences in the length distributions of merged pairs between low- and high-salt fractions (Fig. 2A). The majority of fragments in no-salt and low-salt chromatin was ∼100 bp long, as observed previously (Hasson et al. 2013; Henikoff et al. 2015), whereas the high-salt fraction resulted in a heterogeneous length distribution in which CENP-A-associated fragments ranged from ∼100 to 450 bp. As our original expectation was that centromeres consist of arrays of centromeric nucleosomes (Hasson et al. 2013), we were surprised to observe such size heterogeneity for the bulk of centromeric fragments.

### Solubility of CENP-A/B/C reflects particle size

Next, we analyzed CENP-A, CENP-B, and CENP-C profiles on various α-satellite contigs to investigate the basis for heterogeneous protection. For D7Z1 and D5Z2 contigs, we observed well-phased particles consistent between salt fractions, with weaker phasing in the DXZ1 contig. However, reads from the 500 mM fraction spanned a broader region (extended shoulders over CENP-B boxes) than reads from low-salt fractions (Fig. 2B). The narrowing of the peaks in low-salt fractions may have resulted from disruption of chromatin at the boundaries of CENP-A/B/C due to the action of MNase used in N-ChIP experiments. MNase not only digests away linker DNA but also “nibbles” on free DNA ends and cleaves to a variable extent within nucleosomes (Xi et al. 2011; Mieczkowski et al. 2016; Chereji et al. 2017). MNase also digests RNA, and this might have contributed to the loss of CCAN components by loss of α-satellite RNAs, which are required in *cis* for full occupancy of CENP-A and CENP-C (McNulty et al. 2017). The narrower peaks in low-salt fractions are ∼100 bp long, which suggests cleavages around individual CENP-A nucleosomes. Because the high-salt fraction constitutes >80% of the total centromeric chromatin, the presence of larger fragments suggests that the majority of individual CENP-A nucleosomes is partially protected from MNase by the tightly associated CCAN complex proteins.

### CUT&RUN salt fractionation (CUT&RUN.Salt) releases discrete CENP-A-containing complexes

Although ChIP has been the dominant method for mapping specific protein–DNA interactions for more than three decades, recent reports of ChIP-seq artifacts (Park et al. 2013; Teytelman et al. 2013; Jain et al. 2015) have emphasized the importance of validation using non-ChIP methods (Zentner et al. 2015). Of particular concern for centromere studies is the tendency of MNase, which is used for N-ChIP, to cause nibbling and internal cleavages (Brogaard et al. 2012), leading to uncertainty as to whether particles are fully or partially wrapped (Hasson et al. 2013). We recently introduced CUT&RUN, an efficient targeted nuclease method that is unrelated to ChIP in that it causes precise cleavage and release of intact antibody targeted particles without solubilizing the rest of the genome (Skene and Henikoff 2017b). In our most recent CUT&RUN protocol (Skene and Henikoff 2017a), antibodies are added to permeabilized cells bound to magnetic beads followed by addition of a protein fusion between MNase and protein A (pA-MN), which binds to the antibody. MNase is activated by calcium and then stopped by chelation with EDTA and EGTA in the presence of 175 mM NaCl. When MNase is tethered to specific sites in CUT&RUN, there is no detectable nibbling, accessibility bias, or internal cleavages over a range of more than two orders of magnitude in digestion times even for highly AT-rich DNA. Moreover, because there is no chromatin solubilization, the CUT&RUN cleavage pattern of DNA extracted from the insoluble pellet can also be profiled (Skene and Henikoff 2017b). To adapt CUT&RUN for salt fractionation (CUT&RUN.Salt), chelation stop buffer was added without RNase, and, after removing the supernatant, we incubated the cell/bead pellet with 500 mM NaCl. We then extracted DNA from the low-salt and high-salt supernatants and the final pellet (Supplemental Fig. 1A). CUT&RUN is well suited for salt fractionation in that antibody recognition occurs before the DNA is cleaved, whereas in ChIP, antibody recognition or DNA recovery might be affected by changes in salt-induced particle conformation, such as loss of particle integrity. For all three fractions, we observed a clear enrichment of centromeric α satellites in qPCR assays on DNA from CENP-A, CENP-B, and CENP-C but not in the negative control H3K27me3 CUT&RUN.Salt sequencing libraries (Supplemental Figs. 1B, 2). Consistent with our N-ChIP results, the majority of chromatin (∼70%–80%) was amplified in the high-salt CUT&RUN.Salt fractions (Supplemental Fig. 1C).

When subjected to paired-end 25-bp × 25-bp DNA sequencing and mapped to consensus α-satellite arrays, all three fractions showed strong enrichment for CENP-A, CENP-B, and CENP-C over homogeneous dimeric α satellites (SFI, D5Z2, D7Z1, and SF2) relative to a background control and weak enrichment over noncentromeric α satellites (D5Z1 and D7Z2) (Supplemental Table 1). Pericentric histone marks (H3K9me2 and H3K9me3) showed weak enrichment over α satellites, as expected, whereas euchromatic marks (H3K27me2 and H3K27me3) showed strong depletion.

**Figure 3.**
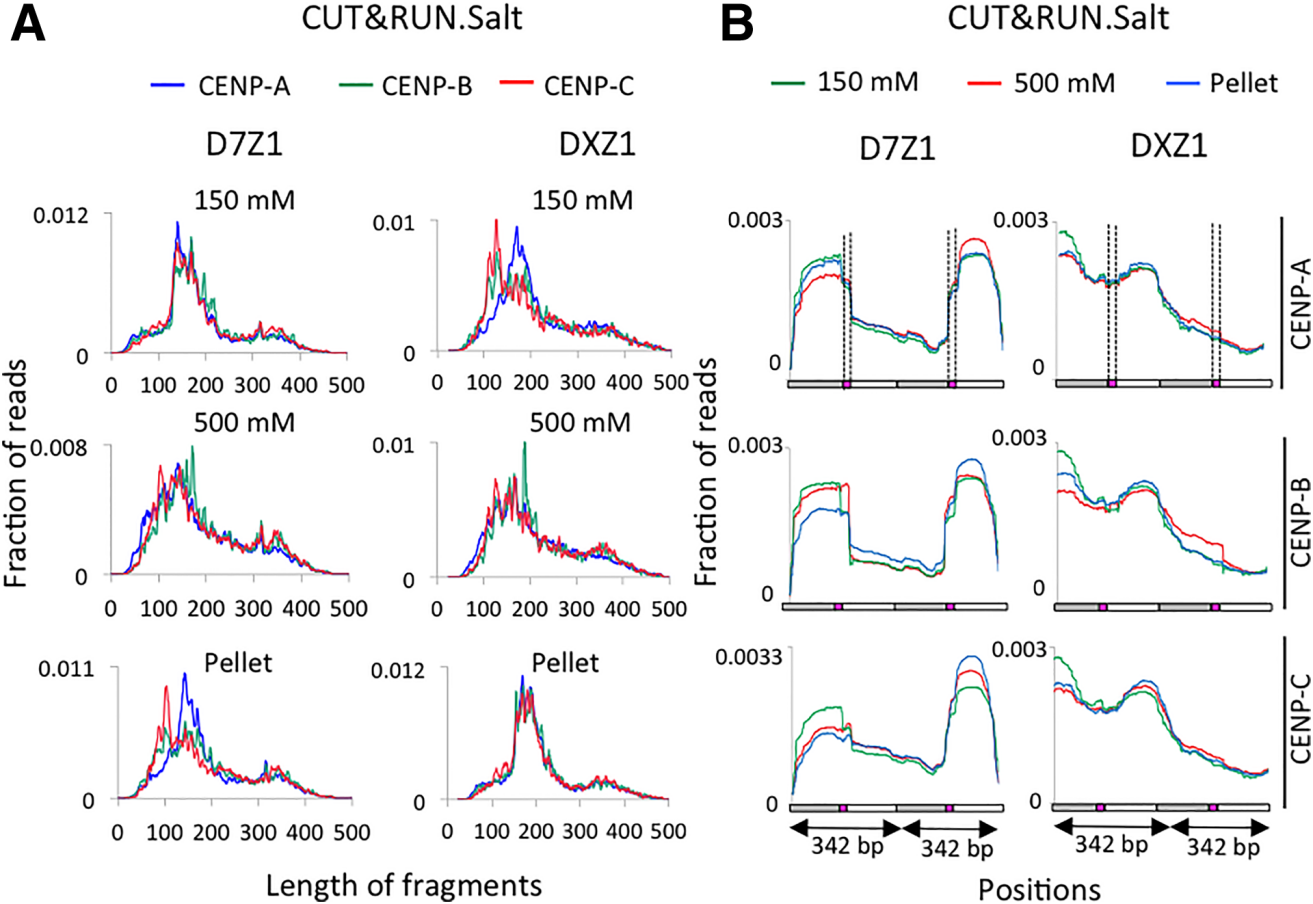
CUT&RUN.Salt releases a discrete CENP-A/B/C complex. (*A*) Fragment length analysis of merged pairs mapped to D7Z1 (*left*) and DXZ1 (*right*) in CENP-A, CENP-B, and CENP-C CUT&RUN.Salt fractions. (*B*) Mapping of CENP-A, CENP-B, and CENP-C CUT&RUN.Salt 250-bp × 250-bp merged pairs to D5Z2, D7Z1, and DXZ1 arrays. A region spanning two tandem dimers from these contigs is presented. Filled boxes represent CENP-B boxes.

To analyze the fragment length distribution of CUT&RUN.Salt fragments, we performed paired-end 250-bp × 250-bp sequencing on CUT&RUN.Salt fractions and mapped merged pairs to active centromeric α-satellite contigs. In contrast to the heterogeneous size distribution seen between N-ChIP salt fractions, we observed much more uniform size distributions between low-salt and high-salt CUT&RUN.Salt fractions (Fig. 3A). For CENP-A, CENP-B, and CENP-C CUT&RUN.Salt, all three fractions showed a major peak at ∼160–185 bp and a minor peak at ∼340 bp. The CENP-A CUT&RUN.Salt profiles on α-satellite contigs revealed discrete CCAN complexes in low-salt, high-salt, and pellet fractions (Fig. 3B) similar to those observed using X-ChIP (Thakur and Henikoff 2016). Thus, CUT&RUN.Salt not only releases the intact CENP-A/B/C complex under native conditions (thereby avoiding potential cross-linking artifacts) but also preserves the particles from disruption, in contrast to N-ChIP, in which untethered MNase produces 100-bp subparticles.

### Strong and dense CENP-B boxes stabilize the CENP-A/B/C complex

**Figure 4.**
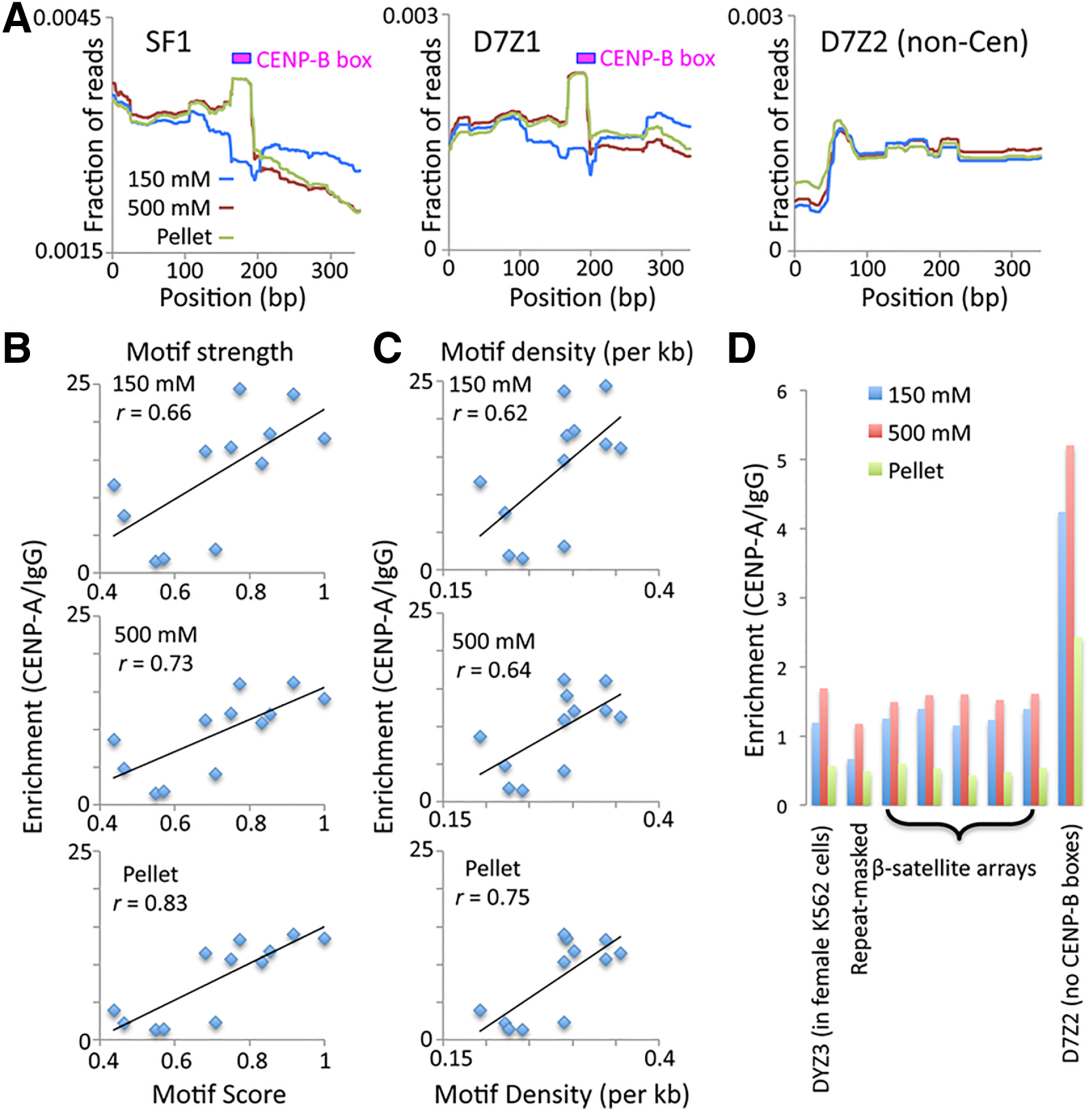
CENP-B stabilizes CENP-A/B/C. CUT&RUN was performed using permeabilized cells (Skene and Henikoff 2017a). (*A*) Mapping of CENP-B CUT&RUN.Salt to SF1, D7Z1, and D7Z2 sequences. To avoid edge effects, paired-end 25-bp × 25-bp reads were mapped to a tandemly triplicated 340-bp dimer consensus sequence representing each contig. The average occupancy over the middle dimer is shown. (*B*) Correlation between CENP-B box motif score (where 1 indicates identity to the central 15 bp of the CENP-B box, and 0 indicates more than three mismatches) and CENP-A/IgG fold enrichment values in CUT&RUN.Salt fractions. The average of two experiments (10-min and 30-min digestion times) is shown. (*C*) Same as *B* for motif density per kilobase. (*D*) CENP-A CUT&RUN.Salt fold enrichments are shown for a Y-chromosome α satellite (DYZ3) that is absent from the female K562 cells used in this experiment, the repeat masked Hg19 genome, annotated β satellites, and α satellites from a homogeneous array (D7Z2) that lacks CENP-B boxes. Data are from 250-bp × 250-bp mapped merged pairs.

As seen above for CENP-A N-ChIP, differential solubility for CENP-A, CENP-B, and CENP-C CUT&RUN.Salt was most evident over the CENP-B boxes, with increasing occupancy seen with increasing salt over the same α-satellite contigs. Interestingly, when averaged over multiple 340-bp units, a peak of CENP-B CUT&RUN occupancy was observed precisely over the CENP-B box in high-salt and pellet fractions but not over the low-salt fraction (Fig. 4A). Taken together with the preservation of CCAN particles in CUT&RUN, this absence of an average peak suggests that there are two distinct classes of particles: stable particles that resist disruption and are enriched for CENP-B and less stable particles that are depleted for CENP-B.

We wondered whether CCAN integrity as measured by CUT&RUN.Salt reflects a stabilizing role of CENP-B. The presence of α-satellite sequences with a gradient of divergence at human centromeres provides an opportunity to test this possibility (Henikoff et al. 2015). The most recently expanded abundant CENP-A-enriched α-satellite dimeric arrays contain a high density of CENP-B boxes (approximately one CENP-B box per 340-bp dimer). Older α satellites become more divergent due to an accumulation of random mutations over evolutionary time, which leads to either complete loss or degeneration of CENP-B boxes. We asked whether the divergence of the CENP-B box sequence from the ancestral motif corresponds to the ability of α satellites to bind CENP-A/B/C and therefore the ability to form centromeres.

To address this question, we first identified the middle 15 bp of the 17-bp CENP-B box as being ancestral, as it is present in the large majority of homogeneous SF1 (e.g., D5Z2), SF2 (e.g., Cen13-like), and SF3 (e.g., DXZ1) α-satellite contigs at regular intervals. We then identified statistically significant occurrences of this motif using motif alignment and search tool (MAST) and scored them between 0 (more than three mismatches) and 1 (identical). We found that the increase in CENP-B motif score correlated with enrichment of CENP-A relative to nonspecific IgG CUT&RUN occupancy (Fig. 4B). Specifically, when averaged over two biological replicates, we observed Pearson correlations of *r* = 0.66–0.83 for all three salt fractions. We conclude that the presence of a strong CENP-B box is associated with stabilization of CENP-A/B/C.

CENP-B box density varies from being highest on the dimeric arrays to the least on heterogeneous monomeric arrays. As CENP-B binds to the CENP-B box in a sequence-dependent manner, CENP-B protein density is also expected to be higher on younger homogenous arrays. We tested whether the degree of loss of CENP-B boxes (decrease in CENP-B box density) from old sequences correlates with the reduction in CENP-A binding on these sequences. We plotted the CENP-B density against CENP-A enrichment on longer α-satellite contigs and observed strong correlations (*r* = 0.62–0.75) between CENP-B motif density and CENP-A enrichment (Fig. 4C). This suggests that maintenance of strong and dense CENP-B boxes increases the efficiency of CENP-A/B/C binding to α-satellite centromeres. Our evidence that CENP-B boxes within homogeneous functional α-satellite arrays have evolved to stabilize the resident CENP-A/B/C particles provides support for the proposal that CENP-B contributes to segregation fidelity by stabilizing CENP-C (Fachinetti et al. 2015).

### Divergent α satellites retain some competence for CENP-A assembly

Although the highest CENP-A enrichment occurred on highly homogenous arrays with dense CENP-B boxes, qPCR assays also revealed a low amount of CENP-A on divergent sequences that contained either sparse or no CENP-B boxes in CUT&RUN.Salt and salt fractionation N-ChIP experiments (Fig. 1C). Detecting low levels of CENP-A cytologically on divergent α satellites is difficult due to their low copy number when compared with the detection of homogenous dimers that are brightly stained with CENP-A. For example, homogenous D7Z1 (1.5–3.8 Mb) shows a strong cytological colocalization with CENP-A, whereas divergent D7Z2 (0.1–0.5 Mb) was reported to be negative for CENP-A binding (Slee et al. 2012). We compared the CENP-A enrichment in CUT&RUN.Salt samples on heterogeneous monomeric α satellites with noncentromeric sequences, including β satellites—a 68- to 69-bp pericentric tandem repeat array. We found more than threefold CENP-A enrichment on D7Z2 relative to the repeat-masked genome and to β-satellite arrays (Fig. 4D), indicating that even a divergent α-satellite array that completely lacks CENP-B motifs retains some competence for CENP-A assembly.

### Unexpected structural and conformational variations of CENP-A/B/C on nearly homogenous α-satellite arrays

**Figure 5.**
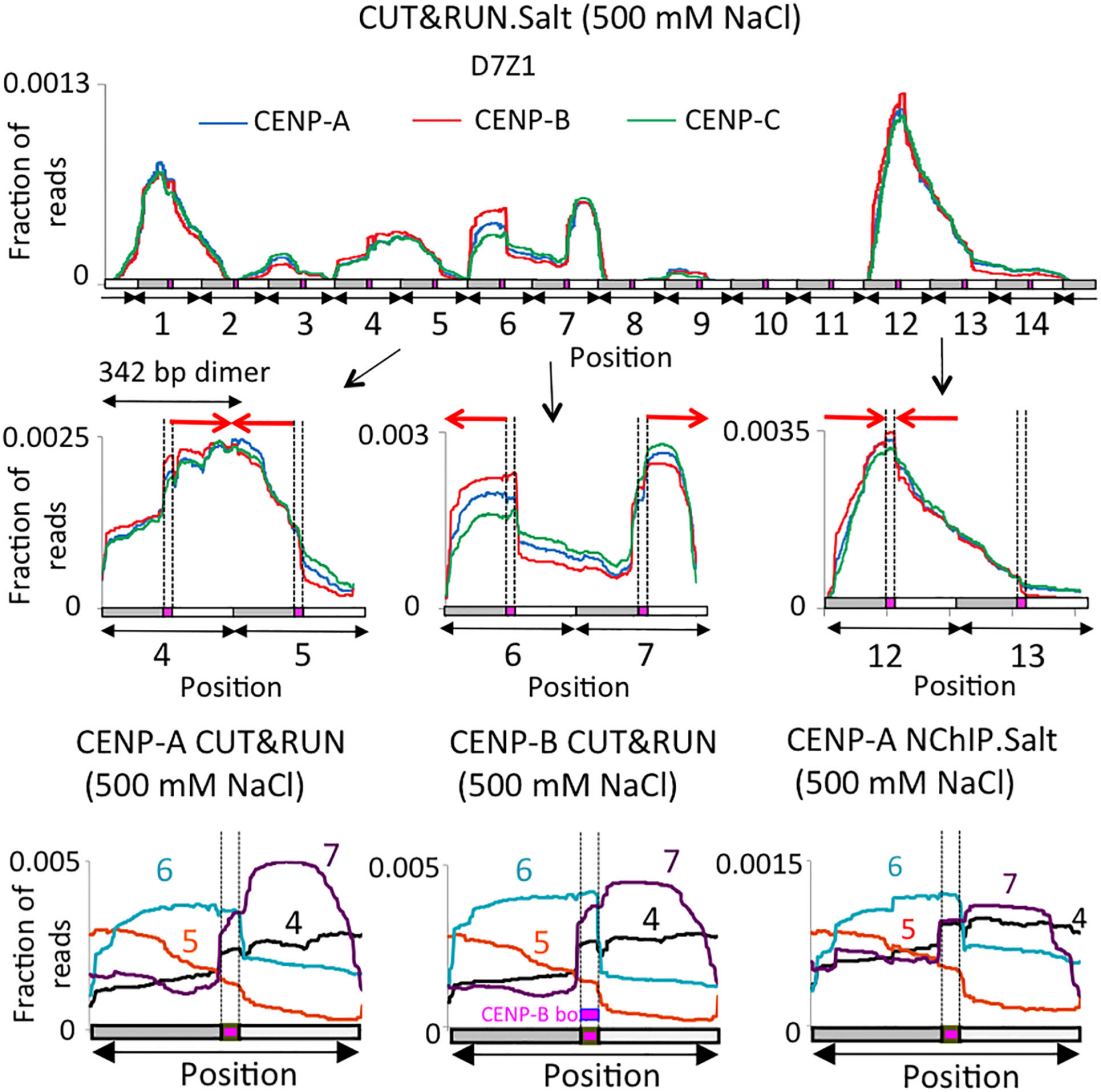
Structural and conformational variations of CENP-A/B/C at human centromeres. (*Top* panels) High-stringency mapping of the CENP CUT&RUN.Salt 250-bp × 250-bp merged pairs averaged over D7Z1. CENP CUT&RUN.Salt profiles of two tandem dimers are shown *below* the D7Z1 contig. (*Bottom* panels) Overlaying of CENP-A profiles from different dimeric units to show the orientation of CENP-A/B/C in either direction in CENP-A and CENP-B CUT&RUN.Salt as well as CENP-A N-ChIP.

Although perfectly homogeneous α-satellite arrays cannot be uniquely assembled from standard sequencing reads, ∼5% divergence is enough to assemble some sequenced reads into contigs. As we had expected that all copies of highly homogeneous arrays would show identical patterns, we were surprised to find major differences between adjacent repeats when we mapped 250-bp × 250-bp merged pairs to them. We observed three major types of variations within homogeneous arrays corresponding to annotated BAC clones and genomic contigs (Fig. 5; Supplemental Fig. 3): (1) Differential occupancy of individual dimers by CENP-A/B/C. We observed up to ∼50-fold differences in enrichment between the lowest and the highest occupied dimers within a single array. (2) Orientation of CENP-A/B/C with respect to the CENP-B box. The distance between two CENP-B boxes within an SF1 α-satellite dimeric array is 340 bp unidirectionally oriented in a head-to-tail fashion. Thus, the orientation of the CENP-A/B/C-containing complex is expected to be unidirectional. Contrary to this expectation, we observed that CENP-A/B/C could be oriented on either side with respect to the CENP-B box orientation (red arrows in Fig. 5) even within a single continuous α-satellite contig. (3) Structural variation. We observed different configurations of CENP-A/B/C on these contigs, including either a symmetric complex spanning the entire 340-bp dimer with almost equal CENP-A/B/C binding on both monomers of the dimer or an asymmetric complex preferentially occupying one monomer of the dimer. Such drastic structural variations of CENP-A-containing particles on α-satellite dimers were observed with remarkably little difference in sequence. For example, the four adjacent 340-bp D7Z1 repeat units shown superimposed in the bottom panels of Figure 5 are 88%–96% identical in pairwise comparisons, and yet all four are different from one another in CCAN structure. Thus, it would appear that slight α-satellite sequence variations affect the binding behavior of CENP-A-containing complexes. Evidently, multiple CCAN forms can recruit the outer kinetochore, although it is possible that only a single structural form is competent for recruitment. These differences could be inherent to the sequences to which the CCANs are bound or reflect exclusion by nonhistone satellite DNA-binding proteins analogous to *Drosophila* D1, GAGA factor, and Prod proteins (Levinger and Varshavsky 1982; Raff et al. 1994; Torok et al. 1997).

Our mapping of CENP-A/B/C using salt fractionation confirms our previous report in which we showed that homogeneous α-satellite arrays are occupied by a single coherent CCAN complex containing CENP-A, CENP-B, CENP-C, and CENP-T (Thakur and Henikoff 2016). Our mapping of the CENP-T subcomplex over the CENP-B box led us to propose a model in which each α-satellite dimeric unit wraps with right-handed superhelical chirality around the CENP-TWSX subparticle between two CENP-A/H4/H2A/H2B subnucleosomes. The sensitivity of the uncross-linked CCAN to MNase digestion can account in part for the differences in DNA protection that led to conflicting conclusions about the structure of the CENP-A nucleosome. However, by following N-ChIP with salt fractionation, we now show that CENP-A particles observed using low-salt conditions (Lacoste et al. 2014; Nechemia-Arbely et al. 2017) comprise only a minor fraction of the total CENP-A genome-wide. In contrast, the major N-ChIP salt fraction consists of particles that protect much larger DNA fragments, consistent with the presence of an intact CCAN complex. Our evidence that CENP-B binding to CENP-B boxes in homogeneous α-satellite arrays promotes CCAN integrity provides evidence for a specific role for CENP-B. In addition, our finding that CCAN components are recruited at low levels to the D7Z2 α-satellite array that lacks CENP-B boxes and shows no enrichment of CENP-B suggests that there is inherent CCAN recruitment potential even in the absence of CENP-B. Thus, CCAN occupancy is determined by α-satellite sequence but can be enhanced by CENP-B binding to arrays.

## Materials and methods

### Cell lines, antibodies, and primers

Salt fractionation N-ChIP assays were performed in the CENP-A Flag-tagged HT1080-1b cell line (Thakur and Henikoff 2016), and CUT&RUN.Salt experiments were performed in the K562 cell line. The antibodies used were anti-CENP-A (Abcam, ab13939), anti-CENP-B (Abcam, ab25734), anti-CENP-C (Abcam, ab33034), Histone H3K27me3 (Cell Signaling Technologies, 9733), IgG (Antibodies Online, ABIN102961) and MTPOL (GeneTex, GTX105137). qPCR primers are listed in Supplemental Table 2.

### N-ChIP-seq with salt fractionation

Nuclei were prepared from HT1080-1b cells under native conditions, digested with MNase as described (Henikoff et al. 2015), and subjected to salt fractionation (Henikoff et al. 2009). Briefly, MNase-digested nuclei were centrifuged at 500*g* for 5 min at 4°C. The supernatant was saved as the no-salt fraction. The pellet was resuspended in Triton buffer I (150 mM NaCl, 10 mM Tris–HCl at pH 7.4, 2 mM MgCl_2_, 2 mM EGTA, 0.1% Triton X-100, 0.5 mM phenylmethylsulfonyl fluoride), incubated for 2 h at 4°C on a shaker, and then centrifuged at 500*g* for 5 min at 4°C. The resulting supernatant was saved as the 150 mM salt fraction. Following the same scheme, the pellet was successively fractionated in Triton buffer I containing 300 and 500 mM NaCl. For subsequent steps, the NaCl concentration of all samples was adjusted to 200 mM to avoid the disruption of antigen–antibody interaction in subsequent steps. NaCl-adjusted fractions were subjected to N-ChIP as described (Henikoff et al. 2015).

### CUT&RUN.Salt

CUT&RUN of human K562 cells or nuclei was performed essentially as described (Skene and Henikoff 2017b) except that, after digestion, the protocol was modified to allow for salt fractionation, as described in the accompanying step-by-step protocol (Supplemental Material). Experiments shown in Figure 4 and Supplemental Table 1 used permeabilized cells rather than nuclei (Skene and Henikoff 2017a). Paired-end 250-bp × 250-bp or 25-bp × 25-bp sequencing was performed by the Fred Hutch Shared Genomics Resource.

### Sequence analysis

Paired-end 250-bp × 250-bp reads were trimmed and merged using SeqPrep with parameters: -q 25 -L 25 -o 15 as described (Henikoff et al. 2015). Merged pairs and paired-end 25-bp × 25-bp reads were mapped using Bowtie2 with following parameters: --end-to-end --very-sensitive --no-mixed --no-discordant -q --phred33 -I 10 -X 700. For CUT&RUN.Salt, read counts were calibrated using the spike-in control as described (Skene and Henikoff 2017b). Enrichment values represent the ratio of calibrated read counts for the specific antibody versus a nonspecific IgG control. To estimate motif strength and densities, we reasoned that the 15-bp CENP-B box motif TTCGTTGGAAACGGG is ancestral, as it is found at regular intervals in the most homogeneous SF1 (e.g., Cen1-like), SF2 (e.g., Cen13-like), and SF3 (e.g., DXZ1) α-satellite arrays. We scanned contigs for statistically significant occurrences as described (Zentner et al. 2015) to identify CENP-B motifs and calculate CENP-B box mismatches and density. We define a motif score as the degree of identity to the 15-bp consensus, where 15 out of 15 matches equals 1, more than three mismatches equals 0, and each mismatch subtracts a value of 0.25, for a scale of 0 (no significant motif) to 1 (perfect motif).

### Accession number

Data have been deposited in Gene Expression Omnibus (GSE104805).

## Supplementary Material

Supplemental Material
