# Supplementary material for: Unexpected conformational variations of the human centromeric chromatin complex
Source: Genes Dev. 2018 Jan 1;32(1):20–5. doi: 10.1101/gad.307736.117 (PMC5828391; doi:10.1101/gad.307736.117)
Supplement: Supplemental Material [file supp_32_1_20__index.html]

Unexpected conformational variations of the human centromeric chromatin complex — Supplemental Material 

# Unexpected conformational variations of the human centromeric chromatin complex

## Supplemental Material

- Supplemental\_Material.pdf
- Supplemental\_Fig1.pdf
- Supplemental\_Table1.pdf
- Supplemental\_Fig2.pdf
- Supplemental\_Table2.pdf
- Supplemental\_Fig3.pdf
