# Supplementary material for: Unexpected conformational variations of the human centromeric chromatin complex
Source: Genes Dev. 2018 Jan 1;32(1):20–5. doi: 10.1101/gad.307736.117 (PMC5828391; doi:10.1101/gad.307736.117)
Supplement: Supplemental Material [file supp_gad.307736.117_Supplemental_Material.pdf]

CUT&RUN.salt protocol[1]

Buffers[2]

Nuclear extraction (NE 50 ml)

- 1 ml 1M HEPES-KOH pH 7.9 (20 mM)
- 500 µL 1M KCl (10 mM)
- 12.5 µL 2M Spermidine (0.5 mM)
- 500 µL 10% Triton X-100 (0.1%)
- 12.5 ml 80% Glycerol (20%)
- water to 50 mL
- 1 large Roche complete EDTA-free tablet

Binding (20 ml)

- 400 µL 1M HEPES-KOH pH 7.9 (20 mM)
- 200 µL 1M KCl (10 mM)
- 20 µL 1M $CaCl_2$ (1 mM)
- 20 µL 1M $MnCl_2$ (1 mM)
- water to 20 ml

Wash (100 ml – hold on ice)

- 2 ml 1 M HEPES pH 7.5 (20 mM)
- 3 ml 5 M NaCl (150 mM)
- 25 µL Spermidine (0.5 mM)
- 333 µL 30% BSA (0.1%)
- water
- 2 large Roche complete EDTA-free tablets

Blocking (5 ml – hold on ice)[3]

- 5 ml Wash buffer
- 20 µL 0.5 M EDTA (2 mM)

2XSTOP+ (5 ml – hold on ice)

- 200 µl 5M NaCl (200 mM)
- 200 µL 0.5M EDTA (20 mM)
- 100 µL 0.2M EGTA (4 mM)
- 50 µL NP40 (0.1%)
- 4.45 ml water
- 10 µL glycogen 20 mg/ml (40 µg/ml)
- 5 µL heterologous DNA @10 ng/ml[4] (10 pg/ml)

Low-salt (5 ml – hold on ice)

- 175 µl 5M NaCl (175 mM)

- 100 µL 0.5M EDTA (20 mM)
- 50 µL 0.2M EGTA (4 mM)
- 25 µL Triton X (0.1%)
- 4.64 ml water
- 5 µL glycogen 20 mg/ml (40 µg/ml)

High-salt addition (5 ml – hold on ice)

- 825 µl 5M NaCl (825 mM)
- 200 µL 0.5M EDTA (20 mM)
- 100 µL 0.2M EGTA (4 mM)
- 25 µL Triton X (0.1%)
- 4.29 ml water
- 5 µL glycogen 20 mg/ml (40 µg/ml)

1XSTOP (5 ml – hold on ice)

- 100 µl 5M NaCl (100 mM)
- 100 µL 0.5M EDTA (10 mM)
- 50 µL 0.2M EGTA (2 mM)
- 25 µL NP40 (0.05%)
- 4.75 ml water
- 5 µL glycogen 20 mg/ml (20 µg/ml)

Procedure[5]

1) Lysis

- Harvest fresh culture(s)[6,7] and count cells. The following is for 8 million mammalian cells (10 time-point samples)
- Spin down at 600g 3' 4°C swing-bucket rotor and decant.
- Resuspend in 1 ml NE buffer by gentle pipetting. Cells may be aliquoted and frozen at this point[8]
- Spin down at 600g 3' 4°C in swing-bucket rotor and decant.
- Resuspend in 600 µL NE buffer per 8 million mammalian cells.

2) Bind to magnetic beads[9]

- Gently resuspend Bio-Mag Plus Concanavalin A coated beads (Polysciences, Inc. #86057).
- Withdraw 200 µL bead slurry (for 10 time points at Step 7), and transfer to 800 µL Binding buffer in a 1.7 ml Eppendorf tube.
- Place on a magnet stand and wash twice in 1 ml Binding buffer[10,11].
- Resuspend in 300 µL Binding buffer.
- While *gently* vortexing the nuclei, slowly add the bead slurry.
- Rotate 5-10 min at room temperature.

3) Block

- Place on the magnet stand, allow to clear (~20 s –>2 min) and pull off the liquid.
- Add 1 ml Blocking buffer and mix either with gentle pipetting or invert ~10x.
- Incubate 5'.

4) Bind primary antibody

- Place on the magnet stand and pull off the liquid.
- Add 1 ml Wash buffer, invert ~10x.
- Place on the magnet stand and pull off the liquid.
- Resuspend in 250 µL Wash buffer with gentle pipetting or invert ~10x.
- While *gently* vortexing add 250 µL primary antibody in Wash buffer (typically 1:100).
- Incubate on rotator ≥2 hr at 4°C.
- Quick spin and wash twice in 1 ml Wash buffer[12].

5) Bind secondary antibody (as required)[13]

- Pull off the liquid, and resuspend in 250 µL Wash buffer.
- While *gently* vortexing add 250 µL secondary antibody in Wash buffer (typically 1:100).
- Incubate ≥1 hr on rotator at 4°C.
- Quick spin and wash twice in 1 ml Wash buffer.
- Resuspend in 500 µL Wash buffer.

6) Bind pA-MNase

- Pull off the liquid and resuspend each sample in 250 µL Wash buffer.
- While *gently* vortexing add 250 µL pA-MN in Wash buffer for a final pA-MN concentration of 1:1000 for Batch #5 or 1:400 for Batch #6[14].
- Incubate ≥1 hr on rotator at 4°C.

- Quick spin and wash twice in 1 ml Wash buffer.

7) Digestion

- Pull off the liquid and resuspend in 150 µL Wash buffer per time-point sample (total 0.6 – 1.5 ml).
- Equilibrate to 0°C in ice water or in metal blocks fitted for Eppendorf tubes in ice water.
- Remove a tube from 0°C, add 3 µL 100 mM $CaCl_2$ per 150 µL while vortexing, then return to 0°C.
- Stop each time point at the designated time (5 s to 30 min) with 150 µL 2XSTOP+.

8) Salt fractionation

- Incubate 1 hr 4 ^{o}C to release low-salt CUT&RUN fragments from the nuclear pellet.
- Place on the magnet stand and pull off the liquid to fresh tubes (low-salt fraction).
- Resuspend in 150 µL Low-salt buffer.
- While gently vortexing, carefully add 150 µL High-salt addition buffer dropwise along the side of the tube[15].
- Rotate 1 hr 4 ^{o}C to release high-salt CUT&RUN fragments from the insoluble nuclear chromatin.
- Centrifuge 5' 16,000g.
- Place on the magnet stand and pull off the liquid to fresh tubes (high-salt fraction).
- Resuspend beads in 300 µL 1X STOP buffer (pellet fraction).

9) Extractions

- Low-salt fractions: Add 20 µL 5M NaCl to bring up to 500 mM.
- All fractions: Add 1.5 µL RNAse A (Thermo 10 mg/ml) and incubate 20 min at 37 ^{o}C.
- To each sample add 3 µL 10% SDS (to 0.1%), and 2.5 µL Proteinase K (20 mg/ml).
- Mix by inversion and incubate 10 min 70°C.
- Add 300 µL buffered phenol-chloroform-isoamyl solution (25:24:1) and vortex.
- Transfer to a phase-lock tube, and spin 5 min full speed.
- Add 300 µL chloroform and invert ~10x to mix.
- Remove aqueous to a fresh tube containing 2 µL of 2 mg/ml glycogen.
- Add 750 µL 100% ethanol and mix by vortexing or tube inversion.
- Chill on ice, spin 10 min full speed 4°C.
- Pour off the liquid and drain on a paper towel.
- Wash the pellet (hardly visible) in 1 ml 100% ethanol, spin briefly full speed.
- Carefully pour off the liquid and drain on a paper towel. Air dry.
- When the pellet is dry, dissolve in 25-50 µL 0.1xTE8 (1x = 10 mM Tris, 1 mM EDTA pH8).

10) Optional Ampure bead selection (bead pellet fraction only)

- After chloroform extraction, remove aqueous from the phase-lock tube to a fresh tube.
- Add 150 µL Beckmann Agencourt AMPure XP beads, pipetting 10x up/down or while gently vortexing[16].

- Let sit 10 min, and place on a magnet stand to clear.
- Transfer the liquid to a fresh tube (to remove the remaining beads).
- Add 1 ml ethanol, and mix by vortexing or tube inversion.
- Chill on ice, spin 10 min full speed 4°C.
- Pour off the liquid and drain on a paper towel.
- Wash the pellet (hardly visible) in 1 ml 100% ethanol, spin briefly on full.
- Carefully pour off the liquid and drain on a paper towel.
- When the pellet is dry, dissolve in 25-50 µL 0.1xTE8.

11) Prepare DNA sequencing libraries[17]

- Optional: Quantify (*e.g.*, 1 µL in Qubit), and/or resolve (*e.g.*, 2 µL in Tapestation)[18].
- Follow the protocol described in PMID:23139805 (without fragmentation or size selection)[19] to make libraries for *paired-end* Illumina sequencing[20].
- Map paired-end reads to the experimental and spike-in calibration genomes[21].

Notes and tips

1. This protocol updates Appendices 2 and 3 of PJ Skene & S Henikoff (2017) *eLife* https://elifesciences.org/content/6/e21856.
2. Buffers should be held on ice. Except as noted, all steps may be performed at room temperature.
3. After the cells bind to the beads, an EDTA-containing blocking solution removes excess divalent cation used to activate the ConA, because carry-over of Ca^{++} from the beads can prematurely initiate strand cleavage after addition of pA-MN. Spermidine in the wash buffer is intended to compensate for removal of Mg^{++}, which might otherwise affect chromatin properties.
4. Heterologous spike-in DNA should be fragmented down to ~200 bp mean size, for example an MNase-treated sample of mononucleosome-sized fragments. As we use the total number of mapped reads as a normalization factor only, very little spike-in DNA is needed. For example, addition of 1.5 pg results in 1,000-10,000 mapped spike-in reads for 1-10 million mapped experimental reads (in inverse proportion). An aliquot of yeast (or Drosophila) DNA from MNase-treated nucleosomes that can be used as a heterologous spike-in control will be included with the pA-MN aliquot that we are sending out upon request.
5. When applying salt fractionation to digitonin-permeabilized cells, follow the protocol presented in https://www.biorxiv.org/content/early/2017/09/24/193219 through the digestion step, then continue with Step 8 above.
6. CUT&RUN can be performed on formaldehyde cross-linked cells using the Total DNA extraction option (*e.g.* Figure 5D of our *eLife* 2017 paper). Use a detergent cocktail to permeabilize the cells to allow antibody and pA-MN to gain access (1% Triton X-100 and 0.05% SDS in the wash and blocking buffers), However, cross-linking slows digestion, so 30 min 0°C digestion in Ca^{++} is recommended.
7. Our NE buffer lysis procedure has been tested on human and Drosophila cultured cells. But because nuclei isolation procedures vary based on organism, cell type and tradition, starting

at Step 2 with native (fresh or frozen) nuclei as we do with yeast is a suitable alternative, as long as care is taken to avoid DNA damage by breakage or apoptosis.

8. For CUT&RUN without magnetic beads, perform wash steps by centrifugation for 3' at 600g in a swing-bucket rotor and careful decanting as described in Appendix 3 of our *eLife* 2017 paper.
9. Maintaining DNA integrity in CUT&RUN is crucial, and we have found that the flash-freezing mammalian cells in glycerol can result in increased background cleavages relative to using fresh cells, similar to to [what has been observed for ATAC-seq](). We recommend cryopreservation in 10% DMSO using a [Mr. Frosty isopropyl alcohol chamber](), which minimizes background degradation.
10. Before placing a tube on the magnet stand, a very quick spin on a mini-centrifuge (e.g. no more than 100 x g for one second) will remove liquid adhering to the cap and sides without crushing the nuclei.
11. A sturdy magnet stand is highly recommended for clean separations. For example, the MACSiMAG Separator (Miltenyi Biotec) requires ~10-30 seconds to clear and allows clean removal of the liquid from the bottom of 1.7 ml Eppendorf tubes.
12. For each wash step: After pulling off the liquid, remove the tube from the magnet stand, add Wash buffer, invert the tube(s) (or gently pipette up/down) until beads release from the tube surface (some clumping is normal), quick spin (≤ 100 x g), and replace on the magnet stand to clear.
13. The binding efficiency of Protein A to the primary antibody depends on host species and IgG isotype (for example see here https://www.thermofisher.com/us/en/home/references/molecular-probes-the-handbook/tables/binding-profiles-of-protein-a-and-protein-g.html). A secondary antibody can be added as an adapter, for example, rabbit anti-mouse after a mouse primary antibody.
14. We will FedEx aliquots of purified pA-MNase protein at 140 µg/ml (Batch #6) in 50% glycerol upon request. We have not observed any loss of activity after several months at –20°C, or after more than a week at room temperature. We find that a 1:400 final concentration of pA-MN Batch #6 for 200 µL of bead slurry in a 500 µL volume is enough to saturate IgG, even for abundant epitopes such those on histones.
15. Nuclei will lyse during this step and release their viscous contents resulting in a stringy precipitate. This is normal. However, too rapid addition of high-salt addition buffer can cause a large blob to form which might impede release of the high-salt-soluble chromatin.
16. For the AMPure bead size selection, you are separating short fragments from very large high MW DNA. As such, this mixture is very "gloopy" and easiest to pipette with cut-off tips.
17. Paired-end 25x25 bp sequencing is sufficient for large mammalian genomes. Separation of sequenced fragments into ≤120 bp and ≥150 bp size classes provides mapping of the local vicinity of a DNA-binding protein, but this can vary depending on the steric access to the DNA by the tethered MNase. Single-end sequencing is not recommended for CUT&RUN, as it sacrifices resolution and discrimination between transcription factors and neighboring nucleosomes. Top of Form
18. The soluble DNA recovered is too low in amount and too small to be detected by gel analysis, and for typical transcription factors the amounts are also too low to be detected by

Tapestation (or equivalent) analysis, and in general it is necessary to make a library to quantify, either by Tapestation analysis or Illumina sequencing. However, using a histone epitope, it is possible to detect and quantify CUT&RUN DNA by Tapestation analysis. An example is shown for H3K27me3 from K562 cells using this protocol. By using this as a quantitative positive control, the likely success of an experiment can be determined without the need to construct and sequence libraries.

19. See Christine Codomo's step-by-step protocol for preparing Illumina libraries from available reagents. We follow this protocol for preparing all of our CUT&RUN libraries.
20. We have also obtained good results with the KAPA hyper-prep kit (https://www.kapabiosystems.com/product-applications/products/next-generation-sequencing-2/dna-library-preparation/kapa-hyper-prep-kits/). Rapid PCR cycles favor exponential amplification of the desired CUT&RUN fragments over linear amplification of intervening fragments that are too long for polymerase to completely transit. Therefore, the number of PCR cycles should be at least 12 cycles, preferably with a 10 second 60°C combined annealing/extension step. This is especially important when using the Total DNA protocol, because AMPure bead size selection only partially removes the undesirable large fragments.
21. We align paired-end reads using Bowtie2 version 2.2.5 with options: --local --very-sensitive-local --no-unal --no-mixed --no-discordant --phred33 -I 10 -X 700. For mapping spike-in fragments, we also use the --no-overlap --no-dovetail options to avoid cross-mapping of the experimental genome to that of the spike-in.
