# Supplementary figures and images for: Unexpected conformational variations of the human centromeric chromatin complex
Source: Genes Dev. 2018 Jan 1;32(1):20–5. doi: 10.1101/gad.307736.117 (PMC5828391; doi:10.1101/gad.307736.117)

Supplementary figure1

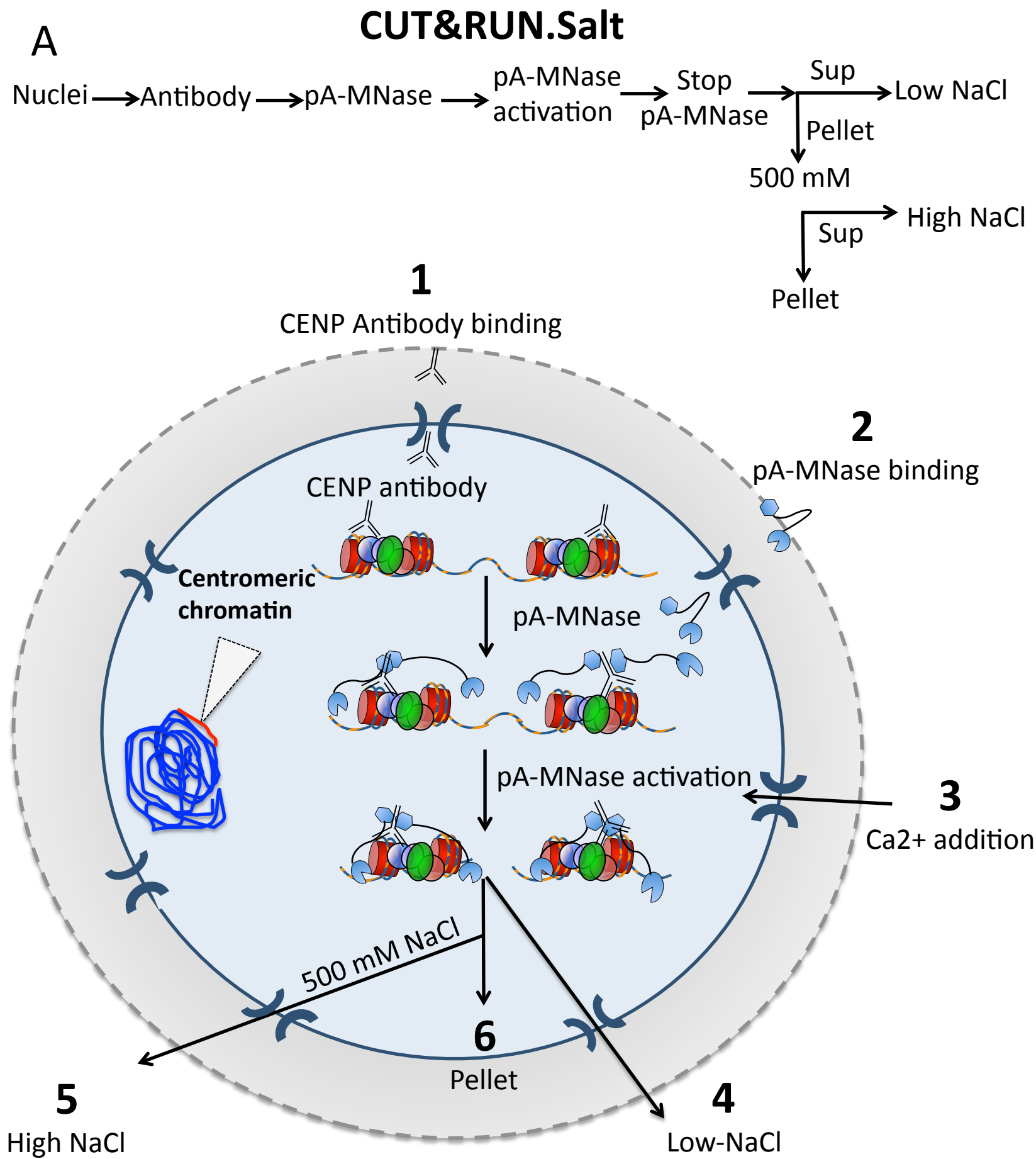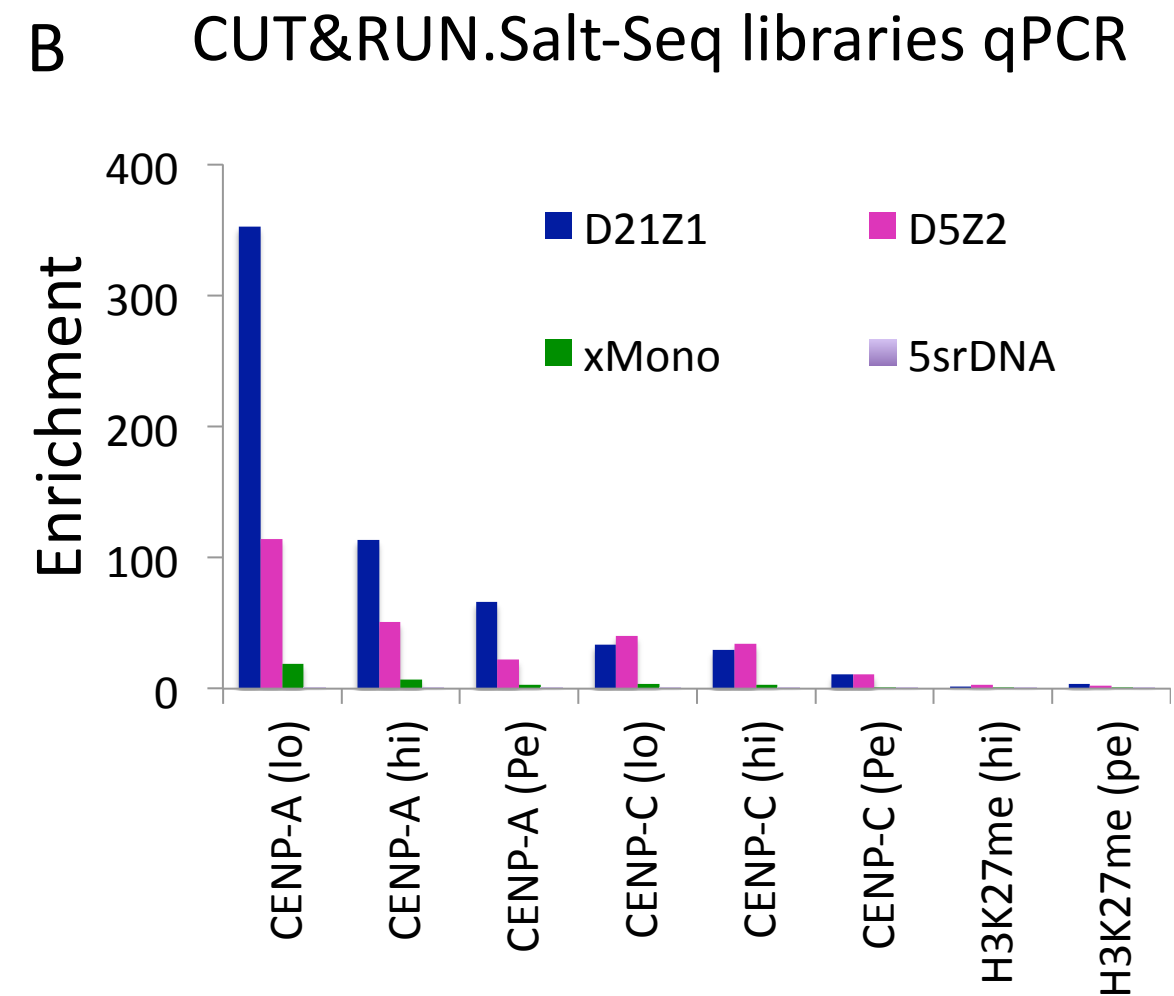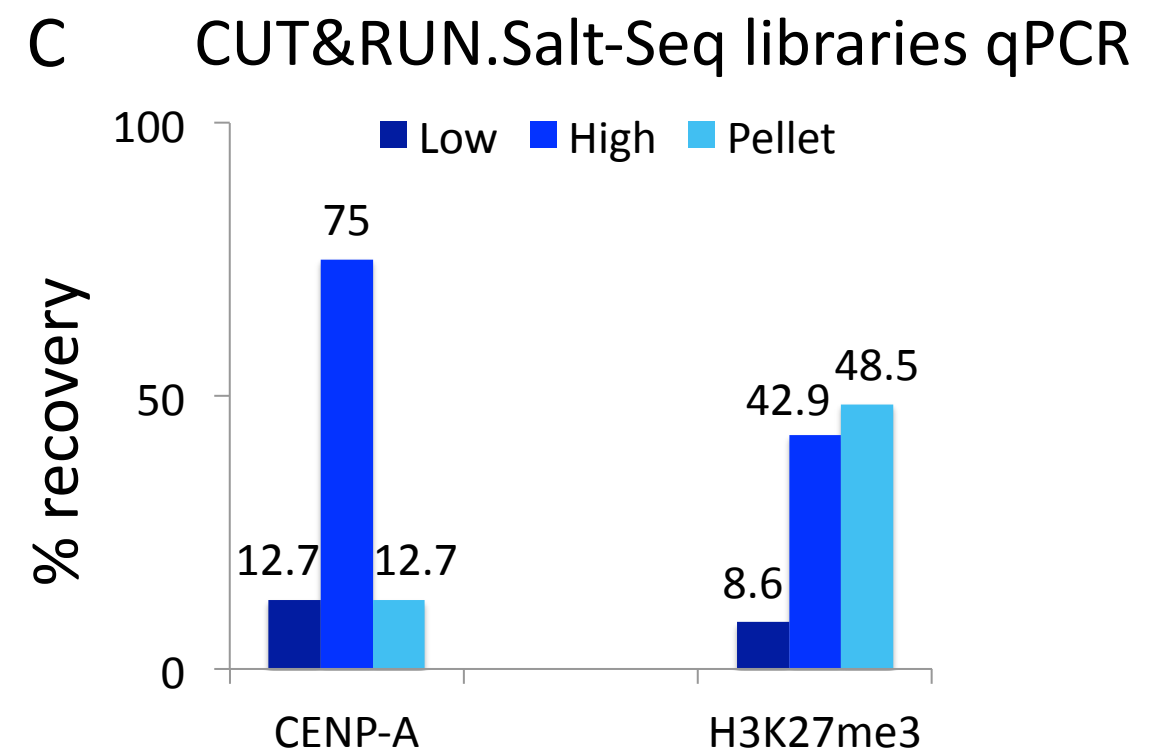

Supplement: Supplemental Material [file supp_gad.307736.117_Supplemental_Fig1.pdf]

Supplementary figure2

CENP-B CUT&RUN.Salt

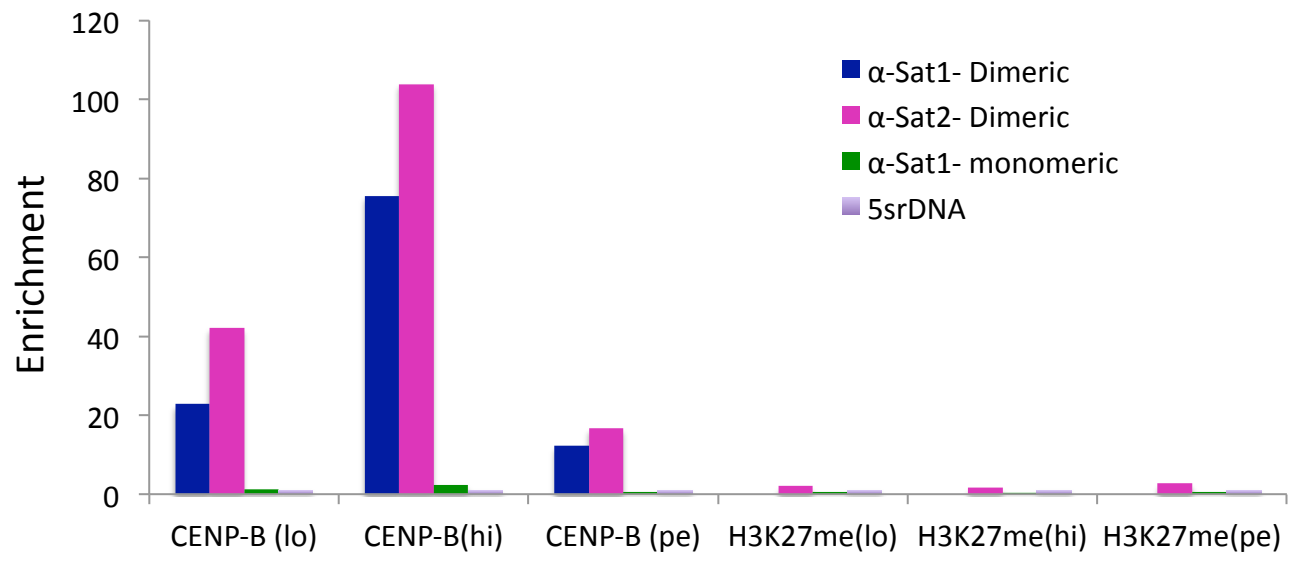

CENP-B CUT&RUN.Salt

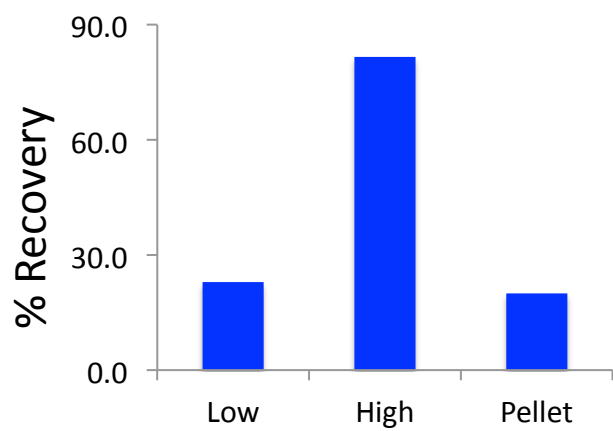

Supplement: Supplemental Material [file supp_gad.307736.117_Supplemental_Fig2.pdf]

Supplementary Figure 3

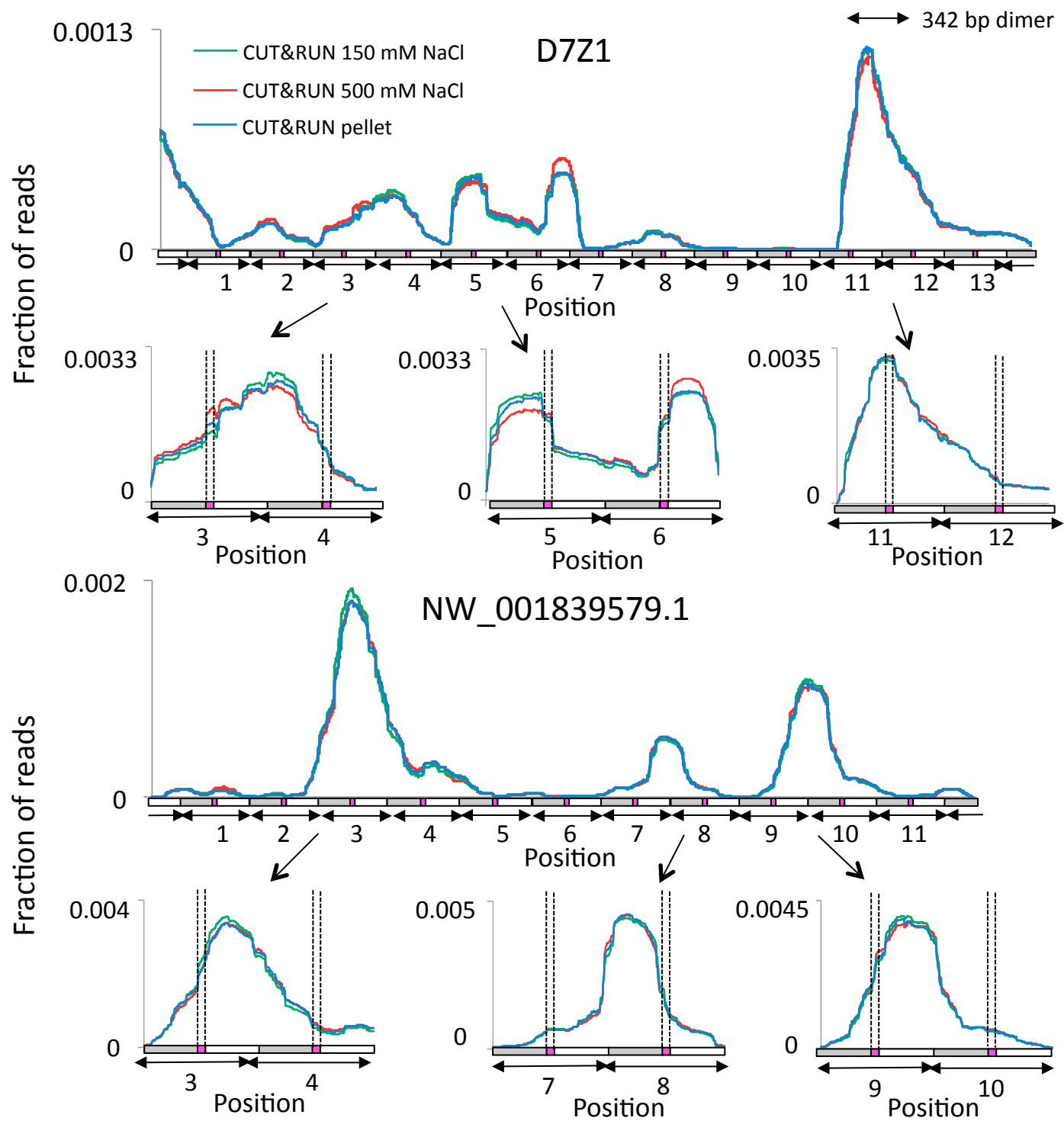

Supplement: Supplemental Material [file supp_gad.307736.117_Supplemental_Fig3.pdf]
