# Supplementary material for: Unexpected conformational variations of the human centromeric chromatin complex
Source: Genes Dev. 2018 Jan 1;32(1):20–5. doi: 10.1101/gad.307736.117 (PMC5828391; doi:10.1101/gad.307736.117)
Supplement: Supplemental Material [file supp_gad.307736.117_Supplemental_Table1.pdf]

Supplementary Table 1. CUT&RUN.salt percent enrichment on α-satellites*

Low-salt	CENP-A	CENP-B	CENP-C	K9me2	K9me3	MTPOL	K27Ac	K27me3
**SF1**	9.99	7.88	3.89	1.13	0.89	0.53	0.06	0.1
**D5Z2**	10.65	8.18	4.09	1.18	0.93	0.56	0.06	0.11
**D7Z1**	6.39	3.91	2.43	0.64	0.56	0.36	0.04	0.05
**SF2**	2.82	1.24	1.04	0.18	0.22	0.12	0.01	0.02
**DXZ1**	0.82	0.35	0.35	0.12	0.07	0.05	0.01	0.03
**D19Z1**	1.39	0.58	0.46	0.17	0.16	0.09	0.01	0.02
**Xmono**	1.46	0.68	0.54	0.17	0.22	0.13	0.02	0.02
**D7Z2**	0.74	0.42	0.21	0.09	0.11	0.05	0.01	0.01
**D5Z1**	0.61	0.32	0.23	0.08	0.08	0.04	0.01	0.01
**Total**	34.9	23.6	13.2	3.8	3.2	1.9	0.2	0.4
**High-salt**								
**SF1**	4.44	11.43	6.16	0.89	0.75	0.53		
**D5Z2**	4.72	11.35	6.46	0.93	0.78	0.56		
**D7Z1**	2.93	6.36	4.01	0.51	0.47	0.36		
**SF2**	1.11	1.63	1.59	0.15	0.17	0.12		
**DXZ1**	0.39	0.42	0.51	0.09	0.06	0.05		
**D19Z1**	0.56	0.6	0.62	0.12	0.12	0.09		
**Xmono**	0.65	0.77	0.72	0.14	0.18	0.13		
**D7Z2**	0.3	0.37	0.32	0.07	0.08	0.05		
**D5Z1**	0.28	0.33	0.33	0.06	0.06	0.04		
**Total**	15.4	33.3	20.7	3	2.7	1.9		
**Pellet**								
**SF1**	6.63	4.03	2.59	0.86	0.68	0.53		
**D5Z2**	4.72	11.35	6.46	0.93	0.78	0.56		
**D7Z1**	4.47	2.38	1.57	0.51	0.44	0.36		
**SF2**	1.11	1.63	1.59	0.15	0.17	0.12		
**DXZ1**	0.59	0.17	0.2	0.08	0.06	0.05		
**D19Z1**	0.56	0.6	0.62	0.12	0.12	0.09		
**Xmono**	0.95	0.34	0.25	0.15	0.15	0.13		
**D7Z2**	0.45	0.11	0.08	0.06	0.06	0.05		
**D5Z1**	0.41	0.13	0.11	0.06	0.05	0.04		
**Total**	23.2	12.1	8.4	2.9	2.4	1.9		

*PE25x25 reads were mapped to tandemly triplicated consensus sequences. The percentage of the total number of reads mapping to the hg38 human genome assembly for each α-satellite array and for column totals are shown. K9me2 and K9me3, respectively di- and tri-methylated Histone H3K9, are examples of pericentric marks, and K27ac and K27me3, respectively acetylated and trimethylated histone H3K27, are examples of euchromatic marks. The MTPOL antibody against mitochondrial RNA was used as a background control.
