# Supplementary material for: Unexpected conformational variations of the human centromeric chromatin complex
Source: Genes Dev. 2018 Jan 1;32(1):20–5. doi: 10.1101/gad.307736.117 (PMC5828391; doi:10.1101/gad.307736.117)
Supplement: Supplemental Material [file supp_gad.307736.117_Supplemental_Table2.pdf]

Supplementary Table 1. Primers used for qPCR in this study.

Primer	Sequence
Dimeric-α-Sat1F	AGGGAATGTCTTCCCATAAAAACT
Dimeric-α-Sat1R	GTCTACCTTTTATTTGAATTCCCG
Dimeric-α-Sat2F	TTTTTGTGCAATTGGCAAATGGAG
Dimeric-α-Sat2R	AGACTGTTTCCTCACTGCTCT
Monomeric-α-SatF	TTCAACTCACAGAGTTGTACC
Monomeric-α-SatR	GCACTACAAATATCCACCTGC
5srDNA-F	CCGGACCCCAAAGGCGCACGCTGG
5srDNA-R	TGGCTGGCGTCTGTGGCACCCGCT
